# Disentangling Woodland Caribou Movements in Response to Clearcuts and Roads across Temporal Scales

**DOI:** 10.1371/journal.pone.0077514

**Published:** 2013-11-05

**Authors:** David Beauchesne, Jochen AG. Jaeger, Martin-Hugues St-Laurent

**Affiliations:** 1 Department of Geography, Planning and Environment & Centre for Northern Studies, Concordia University, Montréal, Canada; 2 Department of Geography, Planning and Environment, Concordia University, Montréal, Canada; 3 Département de Biologie, Chimie et Géographie, Centre for Northern Studies & Centre for Forest Research, Université du Québec à Rimouski, Rimouski, Canada; Università degli Studi di Napoli Federico II, Italy

## Abstract

Although prey species typically respond to the most limiting factors at coarse spatiotemporal scales while addressing biological requirements at finer scales, such behaviour may become challenging for species inhabiting human altered landscapes. We investigated how woodland caribou, a threatened species inhabiting North-American boreal forests, modified their fine-scale movements when confronted with forest management features (i.e. clearcuts and roads). We used GPS telemetry data collected between 2004 and 2010 on 49 female caribou in a managed area in Québec, Canada. Movements were studied using a use – availability design contrasting observed steps (i.e. line connecting two consecutive locations) with random steps (i.e. proxy of immediate habitat availability). Although caribou mostly avoided disturbances, individuals nonetheless modulated their fine-scale response to disturbances on a daily and annual basis, potentially compromising between risk avoidance in periods of higher vulnerability (i.e. calving, early and late winter) during the day and foraging activities in periods of higher energy requirements (i.e. spring, summer and rut) during dusk/dawn and at night. The local context in which females moved was shown to influence their decision to cross clearcut edges and roads. Indeed, although females typically avoided crossing clearcut edges and roads at low densities, crossing rates were found to rapidly increase in greater disturbance densities. In some instance, however, females were less likely to cross edges and roads as densities increased. Females may then be trapped and forced to use disturbed habitats, known to be associated with higher predation risk. We believe that further increases in anthropogenic disturbances could exacerbate such behavioural responses and ultimately lead to population level consequences.

## Introduction

Most species inhabit environments where meeting biological requirements (e.g. foraging, reproductive activities and parental care) must be balanced with local threats to survival [1]. Organisms thus adapt how they use their habitat as a result of their perception of habitat uncertainty (e.g. risk of predator encounters; [2]), often doing so across multiple spatial scales [3]. Typically, species orient habitat use hierarchically, following the hierarchy of factors likely to affect individual fitness [4]. Likewise, individuals may address limiting factors differentially on a temporal basis with respect to changes in biological states of both prey and predators alongside variation in environmental conditions [5]–[7]. Accordingly, numerous species are strongly cued to plant phenology in periods of high energy requirements [8], although remaining particularly vigilant when moving through their habitat in periods of higher vulnerability [9].

Complete avoidance of limiting factors is however a daunting task for individuals inhabiting heterogeneous environments, and limiting factors may consequently need to be addressed at gradually finer spatiotemporal scales [2], [4]. Such responses may be exacerbated in species with extensive movement patterns due to the inclusion of a greater diversity of habitats in their range [10]–[11]. Individuals may thus be compelled to compromise between biological requirements and risk avoidance on a daily basis, potentially at the expense of other biological activities [12].

While risk assessment is typically associated with predation, it nonetheless involves multiple factors that combine to affect levels of uncertainty associated with a given habitat. Some ecologists have proposed that anthropogenic disturbances (i.e. habitat alterations, resource exploitation and human presence and infrastructure; hereafter called disturbances) could trigger behavioural responses that are similar to those elicited by predators [1], thereby combining with natural stressors to impact prey species and increasing habitat uncertainty. We here define habitat uncertainty as factors that induce disturbance stimuli (e.g. predation risk, forage availability and traffic levels), influence perceived risk and trigger behavioural responses in prey species [12]–[14]. Environments affected by expanding human activities impose significant pressures on prey species [15] and increase the likelihood that wildlife found in such habitats modify their fine-scale behaviour [12], [14]. North-American wildlife inhabiting the boreal forest is currently faced with such a scenario and many species are now found in environments with intensified human activities [16]. Of greatest concern, timber harvesting creates early-seral forests and a dense road network that significantly alters the natural structure of the landscape [17]. As a result, the historical natural disturbance regime has been superseded by forestry-related features over the last century [18].

The objective of this study was to evaluate the impacts of disturbances on individual movements of woodland caribou *Rangifer tarandus caribou* (hereafter referred to as caribou), a threatened species throughout North-America [19]. This species offered a unique opportunity to study the fine-scale movements of species typically associated with mature forests yet inhabiting heavily managed environments [20]. Naturally adapted to cope with natural disturbances across their range [21], remnant caribou populations have nonetheless suffered severe constrictions of their range that are strongly correlated to forestry activities [14], [22]–[23]. The early successional forests created by harvesting jeopardizes the anti-predation strategy used by caribou (i.e. spacing out; [24]) by favouring local increases in moose *Alces alces* [25], gray wolf *Canis lupus* [26] and black bear *Ursus americanus* densities [27]. As a consequence, encounter rates between caribou and predators increase [28]–[29], exacerbating caribou vulnerability.

Like other wildlife species, the woodland caribou has been found to respond to limiting factors across multiple spatiotemporal scales, with predation avoidance on the one hand [4], [24] and disturbances on the other [7], [22] influencing large-scale behaviour, while biological requirements are met at finer scales [30]. We however recently showed that disturbance levels observed within our study area possess the potential to compel individuals to remain in areas increasingly altered [31]. Combined with strong range fidelity [32], it seemed reasonable to expect alterations of fine-scale behaviour in response to the presence of disturbances. We therefore expected (A) that individuals would avoid moving through disturbances and crossing clearcut edges and roads. We further anticipated that (B) the immediate landscape contexts would impact the relative probability of individuals crossing through clearcut edges and roads, highlighting a functional response in habitat selection at a fine spatial scale. Finally, we expected that (C1) individuals would avoid disturbances predominantly during periods of higher vulnerability (e.g. calving) and (C2) during the day when uncertainty associated with disturbances is higher, highlighting an important and thus far mostly overlooked temporal variability in caribou response to disturbances.

## Methods

### Ethics Statement

Woodland caribou is recognized as threatened throughout North-America [19], a status that justifies the emergency of understanding more clearly the mechanisms linking anthropogenic disturbances to the species decline. We then captured, collared and released 49 individuals to assess their behavioral responses to disturbances. Our study was carried out in strict accordance with the recommendations of the Canadian Council on Animal Care, and both captures and manipulations of study animals were approved by the Animal Welfare Committee of the Université du Québec à Rimouski (certificate #36-08-67). Captures were conducted on public lands, under the supervision of the Québec government (i.e. *Ministère des Ressources naturelles et de la Faune du Québec*, hereafter MRNF), so no specific permissions were required.

### Study Area

The study area is located in Québec, Canada, and covers approximately 31 000 km^2^ centered on two adjacent sectors north of the Saguenay-Lac Saint-Jean region: Piraube Lake in the north (49°42′–51°00′N, 71°10′–72°09′W) and Portneuf Lake in the south (48°21′–49°45′N, 69°51′–71°12′W). Mean annual temperature in both areas varies between −2.5 and 0.0°C (extremes ranging from −38 to 33°C) and mean annual precipitation around 1000 and 1300 mm, 30–35% of which is snow [33]. Large mammals found in the area are caribou, moose, gray wolf and black bear. The two sectors are distinguished by their dominant forest cover. Black spruce *Picea mariana* with balsam fir *Abies balsamea*, white birch *Betula paperifera*, white spruce *Picea glauca*, trembling aspen *Populus tremuloides* and jackpine *Pinus banksiana* dominate the southern region, while old-growth coniferous forest and open forest with black spruce, balsam fir and jackpine stands dominate the northern area. Prior to our data collection, the southern and northern regions had a logging history that extended over the last 40 and 15 years, respectively. Our study area thus offered a latitudinal gradient of anthropogenic habitat alteration, with the southern region being altered across ∼35% (Portneuf region) and the northern region by 4% of the forested landscape (Piraube region). Similarly, densities of minor roads were generally greater in the south (1.20 km/km^2^) than in the north (0.04 km/km^2^) while no difference was found for major roads (0.04 km/km^2^. vs. 0.05 km/km^2^, respectively). Such an array of environmental conditions consolidates our understanding of the effects of landscape heterogeneity on caribou movements [34].

### Data Collection

We used global positioning systems (GPS) collars (Lotek models 2200L and 3300L, and Telonics TGW-4680) to monitor 49 female caribou between 2004–2010. We programmed these collars to record a location using time intervals of 1, 2, 3, 4 or 6 hours. Females were preferred for this study as their behaviour can strongly influence calf survival [35]–[36]. Individuals were captured periodically using net-gunning to retrieve data, change batteries or remove collars. Collars were also retrieved following failure or death of an individual.

We used the linear segments connecting two consecutive GPS locations (i.e. steps; [37]) to investigate the influence of anthropogenic disturbances on the sequential movements of individual caribou. In order to obtain uniform data, only time steps of 4 hours were retained for the analysis, other time fixes being subsampled or removed from the dataset. Furthermore, movement behaviour may vary spatiotemporally according to changes in behavioural states of organisms and environmental conditions [5], [6], [12], [38]. Explicitly accounting for such variations by dividing analyses into multiple periods allows to control for confounding factors that, when combined, may hide relevant ecological phenomena [38]–[39] and is therefore becoming increasingly recognized in the literature as good practice when studying animal behaviour [40]–[41]. The analysis was thus divided between six annual periods of biological significance for caribou ecology [7]: spring (15 April–14 May), calving (15 May–14 June), summer (15 June–14 September), rut (15 September–14 November), early winter (15 November–21 February) and late winter (22 February–14 April). Furthermore, as daily behaviour may also vary [12], [38]–[39], each period was further divided between day, dusk/dawn and night times [42], resulting in a total of 18 different periods.

Steps were related to a series of features obtained from digitized ecoforest maps provided by the MRNF and updated each year with new natural and anthropogenic disturbances. Minimum mapping unit size was 4 ha for forested polygons and 2 ha for non-forested areas (e.g., water bodies, bogs). For this analysis, disturbance features included clearcut and road types (Table 1). Clearcuts were categorized according to elapsed time since logging: 0–5 year-old clearcuts, 6–20 year-old clearcuts and established regenerating stands (21–40 years old), whereas roads were divided according to their width: major (i.e. primary and secondary roads respectively 35 and 30 m wide) and minor roads (tertiary and quaternary roads respectively 25 and 20 m wide). No paved roads are found in the study area. The proportion of steps located in each type of clearcut was measured to evaluate the relative probability of individuals moving through clearcuts and a quadratic term was included in order to test for non-linear responses. Relative probability of individuals crossing roads and clearcut edges was evaluated using the number of crossings on each step. The landscape context in which females were moving was also suspected to have an influence on caribou behaviour (e.g. higher probability of crossings when density of clearcut edges is greater). The density of clearcut edges and roads was therefore evaluated in buffers around the beginning of each step (i.e. same density for observed and random steps). Buffer size was determined by a constant radius equal to the median of the periodical step length distributions (i.e. spring: 205 m; calving: 132 m; summer: 245 m; rut: 222 m; early winter: 125 m; late winter: 127 m). We used the median as the step length distribution was characterized by a power law distribution. Consequently, less importance was attributed to the longer and less frequent steps and thus more likely to represent distances traveled by females within the time step analyzed. Topography variables were also included (i.e. the mean elevation on the step and the difference between the elevation at the end and the beginning of the step) in the analysis as altitude and slope have been found to be important features influencing the movements of caribou and other ungulates [38], [43].

**Table 1:** Description of variables considered in the conditional logistic regressions explaining caribou relative movement probabilities in relation to disturbances for 49 female caribou in Saguenay – Lac-Saint-Jean (Québec, Canada) between 2004 and 2010.

Group	Variable	Description
Elevation	ElevVar	Elevation difference between beginning and end of the step
(Elev)	ElevMoy	Mean step elevation
Clearcuts	Cut05	Proportion of 0–5 year-old clearcuts under the step
(Cut)	Cut05^2^	Quadratic term for Cut05
	Cut620	Proportion of 6–20 year-old clearcuts under the step
	Cut620^2^	Quadratic term for Cut620
	Regen	Proportion of regenerating stands (21–40 years old) under the step
	Regen^2^	Quadratic term for Regen
Cross_Edge	Cross_05_	Number of 0–5 year-old clearcut edge crossings
(Cr_Ed)	Cross_620_	Number of 6–20 year-old clearcut edge crossings
	Cross_RGN_	Number of regenerating stand (21–40 years old) edge crossings
	Dens_05_	Density of 0–5 year-old clearcut edge around the beginning of the step
	Dens_620_	Density of 6–20 year-old clearcut edge around the beginning of the step
	Dens_RGN_	Density of regenerating stand (21–40 years old) edge around the beginning of the step
Cross_Roads	Roa12	Number of major road (classes 1 and 2) crossings
(Cr_Rd)	Roa34	Number of minor road (classes 3 and 4) crossings
	Dens12	Density of major roads around the beginning of the step
	Dens34	Density of minor roads around the beginning of the step
Dist_Roads (Dt_Rd)	Dvar12	Difference of distance to closest major road between the beginning and end of the step
	Dvar34	Difference of distance to closest minor road between the beginning and end of the step

### Statistical Analysis

The impacts of forest management features on relative movement probabilities were evaluated using a Step Selection Function (SSF; [37]). This method compares use-availability through a conditional logistic regression:
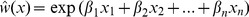
(1)where *β*_1_ to *β*_n_ are coefficients estimated by the regression and *x*_1_ to *x*_n_ are relevant predictors, with higher values indicating greater odds of being selected by an individual. Each observed step was paired with ten random steps originating from the same location and drawn for each individual from unique distributions of step lengths and turning angles (i.e. angle between previous and subsequent location) of all other individuals in order to avoid autocorrelation. Habitat availability thus changed between each step and reflected features immediately available to individuals [38]. Inter-individual and inter-annual variability were also recognized as potential sources of heterogeneity in our data. Such sources of variability could lead a fixed-effect model, which assumes homogeneity in the effects of the independent variables on the dependant variables, to violate the assumption of independence from irrelevant alternatives (IIA hypothesis) [44]. Individuals and years were thus included in the analysis as random terms, constituting a robust safeguard as heterogeneity was suspected yet unknown [44]. Their inclusion also allowed us to control for uneven sample sizes between years and inter-annual and inter-individual variability, while minimizing autocorrelation in the analysis [44]. Autocorrelation between successive steps was further considered by including robust Sandwich estimates of the covariance matrix, which divides observed steps in independent clusters and performs the analysis on the clusters rather than on individual steps (see [37] for details).

A series of candidate models, representing competing hypotheses, was ranked from most to least parsimonious with the quasi-likelihood under independence criterion (QIC), which performs well with conditional logistic regressions [45]. As density measurements were the same for both observed and random steps – and therefore not applicable as fixed factors in a logistic regression – the densities were used solely as interaction terms in the analysis and we tested models with and without interactions to consider the landscape context [46]. Model fit was assessed for each model using a *k*-fold cross validation, which ranks each stratum using the logit values predicted by the logistic regression, with best predictions associated with higher values [38]. A Spearman rank correlation (*r*_s_) was calculated between the ranks and the sum of observed steps in each rank, with strong correlations indicating a propensity for observed steps to be ranked higher. Spearman ranks were averaged over 10 iterations in which model parameters were evaluated using a random 80% of the strata and tested against the remaining 20%. Since most models included in the analysis are nested, inference was based on models with a ΔQIC≤6 [47]. Informative variables explaining relative movement probabilities were then assessed using a confidence interval of 0.95 (i.e. when the 95% CI did not include zero). This enabled us to identify variables that could have been included in the best ranking model without adding significant strength to the model [48]. Data are available from the Dryad Digital Repository: http://dx.doi.org/10.5061 dryad.n3c2f [49].

## Results

A total of 49 female caribou tracked from 1 to 6 years provided 137 867 observed steps (2657±2280 per individual) with numbers varying between each period (7824±3404 per period). Based on the QIC ranking, the best models explaining caribou step selection differed depending on the time of the year and the day (Table 2). The global model was the most parsimonious for 11 of the periods considered, while the global model without interaction prevailed for 4 periods. The remaining 3 periods were best explained by either the proportion of clearcuts under the step or the number of clearcut edge crossings, and partial models that ranked close to the global model (ΔQIC≤6) almost always contained the clearcut variables. This suggests that clearcuts held the most weight in explaining caribou step selection during those periods (Table 2). Validation of best models indicated a high predictive power (r_s_ range from 0.74±0.13 to 0.97±0.03; Table 2).

**Table 2:** Candidate model ranking based on QIC for each period of the day and the year.

Day						
Period	Model structure	K	LL	ΔQIC	ω_i_	r_s_
Spring	Cut	13	−14 549.50	0.00	0.87	0.93±0.05
	Cut+Cr_Ed	19	−14 542.11	5.05	0.07	0.96±0.01
	Cut+Cr_Ed*	16	−14 546.77	5.75	0.05	0.95±0.03
Calving	Cr_Rd+Dt_Rd+Cut+Cr_Ed	25	−16 094.06	0.00	1.00	0.97±0.03
Summer	Cr_Rd+Dt_Rd+Cut+Cr_Ed*	20	−36 134.74	0.00	0.56	0.85±0.08
	Cr_Rd+Dt_Rd+Cut+Cr_Ed	25	−36 126.20	0.46	0.44	0.88±0.05
Rut	Cr_Rd+Dt_Rd+Cut+Cr_Ed*	20	−14 549.51	0.00	0.73	0.74±0.13
	Cr_Rd+Dt_Rd+Cut+Cr_Ed	25	−14 543.53	2.04	0.27	0.84±0.06
Early winter	Cr_Rd+Dt_Rd+Cut+Cr_Ed	25	−15 931.48	0.00	1.00	0.79±0.15
Late winter	Cr_Rd+Dt_Rd+Cut+Cr_Ed	25	−15 459.15	0.00	1.00	0.92±0.06
**Dusk/dawn**						
Spring	Cr_Rd+Dt_Rd+Cut+Cr_Ed*	20	−12 116.71	0.00	0.65	0.95±0.03
	Cr_Rd+Dt_Rd+Cut+Cr_Ed	25	−12 113.68	2.47	0.19	0.93±0.02
	Cut+Cr_Ed*	16	−12 124.81	5.03	0.05	0.94±0.03
	Cut	13	−12 128.91	5.26	0.05	0.93±0.06
	Cut+Cr_Ed	19	−12 122.66	5.74	0.04	0.92±0.03
Calving	Cr_Rd+Dt_Rd+Cut+Cr_Ed	25	−11 280.86	0.00	0.60	0.88±0.10
	Cr_Ed	13	−11 303.92	1.31	0.31	0.88±0.07
	Cr_Rd+Dt_Rd+Cut+Cr_Ed*	20	−11 288.89	4.26	0.07	0.91±0.04
Summer	Cr_Rd+Dt_Rd+Cut+Cr_Ed	25	−27 962.37	0.00	1.00	0.93±0.05
Rut	Cr_Rd+Dt_Rd+Cut+Cr_Ed	25	−16 811.61	0.00	0.99	0.91±0.03
Early winter	Cr_Rd+Dt_Rd+Cut+Cr_Ed	25	−24 922.37	0.00	0.55	0.82±0.06
	Cr_Rd+Dt_Rd	13	−24 936.03	0.58	0.41	0.85±0.09
	Cr_Rd+Dt_Rd+Cut+Cr_Ed*	20	−24 936.74	5.77	0.03	0.85±0.08
Late winter	Cr_Rd+Dt_Rd+Cut+Cr_Ed	25	−15 355.73	0.00	1.00	0.92±0.04
**Night**						
Spring	Cr_Rd+Dt_Rd+Cut+Cr_Ed*	20	−9679.09	0.00	0.68	0.85±0.07
	Cr_Rd+Dt_Rd+Cut+Cr_Ed	25	−9675.25	1.72	0.29	0.85±0.10
Calving	Cr_Ed	13	−6202.93	0.00	0.97	0.75±0.13
Summer	Cut+Cr_Ed	19	−20 418.01	0.00	0.64	0.93±0.03
	Cut+Cr_Ed*	16	−20 422.24	1.38	0.32	0.93±0.04
Rut	Cr_Rd+Dt_Rd+Cut+Cr_Ed	25	−19 502.55	0.00	0.61	0.84±0.14
	Cr_Rd+Dt_Rd+Cut+Cr_Ed*	20	−19 508.29	1.15	0.34	0.83±0.07
Early winter	Cr_Rd+Dt_Rd+Cut+Cr_Ed	25	−34 463.18	0.00	1.00	0.93±0.05
Late winter	Cr_Rd+Dt_Rd+Cut+Cr_Ed	25	−15 891.95	0.00	1.00	0.91±0.06

Models were evaluated using conditional logistic regressions. Only models with ΔQIC≤6 are presented. Number of parameter (K), log-likelihood (LL), difference in QIC values (ΔQIC) and weight (ωi) are given. Model performance was assessed with a Spearman rank correlation (rs±sd). Elevation variables were included in all models tested and models without interactions (i.e. densities of clearcuts edges and roads) are identified with a *.

### Impacts of Clearcuts and Roads on Step Selection

**Figure 1:**
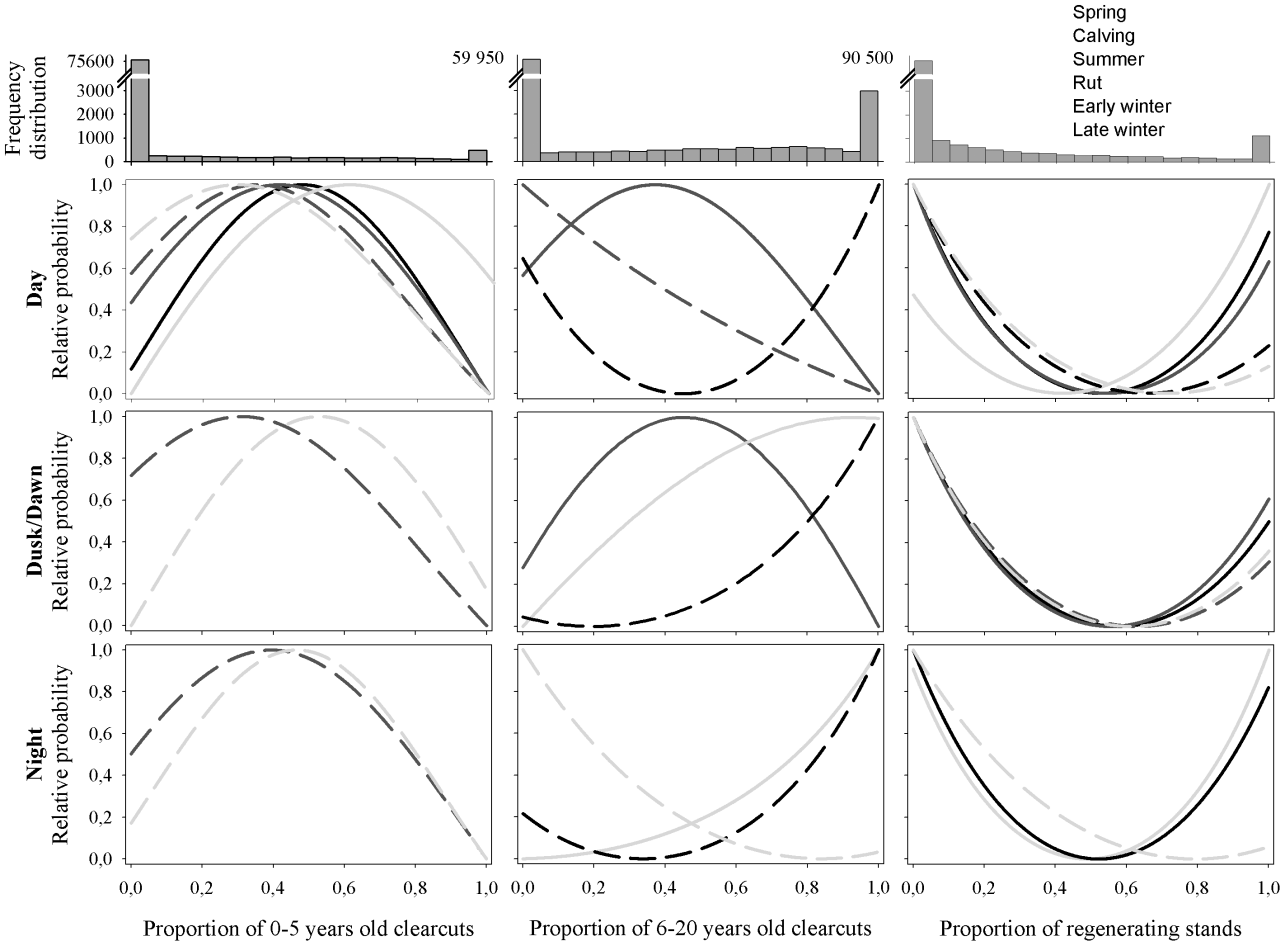
Relative probability of caribou occurrence. Presented as a function of a) the proportion of the step in 0–5 years old clearcuts, b) the proportion of the step in 6–20 years old clearcuts and c) the proportion of the step in regenerating stands for all significant periods. With each graph is associated the annual frequency distribution of the proportion of the step in each clearcut types. The ŵ(x) values obtained through the logistic regression equations were standardized between 0 and 1 to obtain relative probabilities of observing caribou steps.

Caribou mostly avoided clearcuts, using 0–5 year-old clearcuts only in combination with other habitat types and distinctly increasing avoidance as stands aged (Fig. 1; Tables 3–5). Our models predict an increase in the relative probability of caribou occurrence when steps are entirely located in regenerating stands, yet the frequency distributions highlight that such steps have a low probability of being observed within our system (Fig. 1). We thus attributed more weight to the left side of the curves when interpreting our results. Response to clearcuts also differed between annual periods. The relative probability of caribou using disturbances increased in late winter and spring until summer and rut, to subsequently decrease markedly in the winter periods (Fig. 1). Caribou avoided disturbances prominently during the day throughout all periods. Certain types of disturbances (e.g. regenerating stands) and periods (e.g. calving and winter periods) were nonetheless marked with increased avoidance during dusk/dawn and at night, although to a lesser extent (Fig. 1; Tables 3–5). Typically, however, female avoidance of disturbances decreased during dusk/dawn and at night, with females sometimes increasing their use instead (e.g. 6–20 year-old clearcuts during summer and rut).

**Table 3:** Coefficient estimates (ß) and 95% confidence intervals (95% CI) of the independent variables of the most parsimonious models explaining caribou movements for 49 females in Saguenay – Lac-Saint-Jean (Québec, Canada) between 2004 and 2010 during daytime.

Variable	Day					
	Spring	Calving	Summer	Rut	Early winter	Late winter
	ß±95% CI*	ß±95% CI*	ß±95% CI*	ß±95% CI*	ß±95% CI*	ß±95% CI*
ElevVar	−**0.0066±0.0014**	−**0.0069±0.0016**	−0.0007±0.0011	−**0.0049±0.0016**	−0.0015±0.0019	**−0.0094±0.0019**
ElevMoy	0.0024±0.0024	**0.0041±0.0027**	**0.0075±0.0019**	**0.0030±0.0028**	**0.0055±0.0033**	**0.0043±0.0032**
Cut05	**2.3675±1.0132**	**1.2350±0.7574**	**0.9892±0.4715**	0.3857±0.8385	**1.9528±1.8162**	1.1719±1.4769
Cut05^2^	**−2.4745±1.0020**	**−1.4895±0.7478**	**−0.8088±0.4614**	**−**0.4003±0.8518	**−2.7657±1.9438**	**−1.9630±1.5867**
Cut620	0.1359±0.4462	**1.3224±0.6743**	0.1064±0.4417	**−0.6521±0.6360**	**−0.7579±0.5280**	**−**0.1725±0.5638
Cut620^2^	**−**0.0391±0.3974	**−1.7738±0.6633**	**−**0.1536±0.4267	**0.7224±0.5881**	0.0450±0.5180	**−**0.4828±0.5478
Regen	**−1.4788±0.6993**	**−2.1337±0.7400**	**−0.7955±0.4649**	**−1.2733±0.8271**	**−**0.7271±0.7766	**−1.2693±0.8079**
Regen^2^	**1.4014±0.7390**	**1.9552±0.7717**	**0.9527±0.4736**	**0.9661±0.8799**	0.5685±0.8243	**0.8866±0.8549**
Cross_05_	*n/a*	0.0016±0.0334	**0.0444±0.0172**	0.0005±0.0301	**−**0.0848±0.0982	0.0115±0.0559
Cross_05_ *Dens_05_	*n/a*	**0.0058±0.0048**	*n/a*	*n/a*	**0.0162±0.0113**	0.0030±0.0112
Cross_620_	*n/a*	**−0.0525±0.0346**	**−**0.0001±0.0163	**0.0391±0.0203**	0.0152±0.0287	0.0084±0.0276
Cross_620_ *Dens_620_	*n/a*	**0.0059±0.0040**	*n/a*	*n/a*	0.0003±0.0030	**−0.0042±0.0039**
Cross_RGN_	*n/a*	**−0.0435±0.0406**	**−0.0286±0.0232**	**−0.0527±0.0392**	**−**0.0124±0.0473	**−0.0531±0.0465**
Cross_RGN_ *Dens_RGN_	*n/a*	0.0044±0.0057	*n/a*	*n/a*	**−**0.0014±0.0079	**−**0.0005±0.0080
Roa12	*n/a*	0.1171±0.2190	**−**0.1763±0.1991	**−**0.2593±0.2701	**−0.3855±0.3170**	**−0.7106±0.3602**
Roa12*Dens12	*n/a*	0.3360±0.5787	*n/a*	*n/a*	0.1157±0.1625	**0.5426±0.5203**
Roa34	*n/a*	0.0357±0.0368	**0.0267±0.0203**	0.0177±0.0313	**−**0.0168±0.0409	**0.0570±0.0322**
Roa34*Dens34	*n/a*	**0.0201±0.0077**	*n/a*	*n/a*	**0.0168±0.0077**	**0.0151±0.0068**
Dvar12	*n/a*	**−**0.0277±0.0402	**−0.0558±0.0270**	**−0.0395±0.0367**	**−**0.0058±0.0462	**−**0.0248±0.0479
Dvar34	*n/a*	**−0.1490±0.0620**	**−0.0859±0.0409**	**−0.1300±0.0537**	**−0.1101±0.0710**	**−**0.0649±0.0742

* Confidence intervals can be obtained by adding and subtracting the ±95% CI value to its associated β value.

Informative variables were identified with the 95% CI (i.e. not overlapping zero) when available (if not, noted as *‘n/a’*) and are identified in bold letters.

**Table 4:** Coefficient estimates (ß) and 95% confidence intervals (95% CI) of the independent variables of the most parsimonious models explaining caribou movements for 49 females in Saguenay – Lac-Saint-Jean (Québec, Canada) between 2004 and 2010 during dusk/dawn.

Variable	Dusk/dawn					
	Spring	Calving	Summer	Rut	Early winter	Late winter
	ß±95% CI*	ß±95% CI*	ß±95% CI*	ß±95% CI*	ß±95% CI*	ß±95% CI*
ElevVar	**−0.0072±0.0018**	**−0.0053±0.0025**	**−**0.0008±0.0012	**−0.0060±0.0018**	**−0.0034±0.0017**	**−0.0071±0.0021**
ElevMoy	0.0031±0.0032	**0.0065±0.0044**	**0.0092±0.0021**	**0.0042±0.0031**	**0.0037±0.0030**	**0.0063±0.0036**
Cut05	1.0586±1.2864	0.8482±1.0360	0.4518±0.5463	0.2154±0.8591	1.1805±1.4161	**1.9011±1.4682**
Cut05^2^	**−**1.2631±1.2689	**−**1.0137±1.0176	**−**0.2717±0.5416	**−**0.0335±0.8464	**−1.8990±1.5536**	**−1.7948±1.5099**
Cut620	0.0515±0.5787	0.8371±0.8610	**0.6203±0.5324**	**−**0.3162±0.6419	**−**0.1706±0.4214	**−**0.2224±0.5629
Cut620^2^	0.1545±0.5265	**−0.9217±0.8369**	**−**0.3321±0.4959	**0.8097±0.5864**	**−**0.1304±0.4147	**−**0.0537±0.5483
Regen	**−1.0068±0.9710**	**−1.0825±0.9731**	0.1601±0.5133	**−**0.5691±0.8290	**−1.1154±0.6555**	**−1.2405±0.8813**
Regen^2^	0.8723±1.0093	0.9758±0.9913	0.0481±0.5464	0.6502±0.8763	**0.8859±0.6738**	**1.0146±0.9382**
Cross_05_	0.0379±0.0451	0.0485±0.0631	0.0206±0.0320	**−**0.0251±0.0504	0.0168±0.0557	**−**0.0262±0.0517
Cross_05_ *Dens_05_	*n/a*	0.0039±0.0078	**0.0096±0.0054**	0.0067±0.0072	0.0029±0.0083	0.0017±0.0133
Cross_620_	**−**0.0050±0.0232	0.0060±0.0539	**−**0.0085±0.0379	**0.0448±0.0367**	0.0131±0.0272	**−**0.0194±0.0304
Cross_620_ *Dens_620_	*n/a*	0.0053±0.0062	**−0.0059±0.0054**	**−**0.0043±0.0054	**−**0.0005±0.0031	0.0016±0.0041
Cross_RGN_	**−0.0880±0.0652**	**−0.1742±0.0998**	**−0.0747±0.0392**	**−0.1409±0.0678**	**−0.0526±0.0513**	**−0.2021±0.0759**
Cross_RGN_ *Dens_RGN_	*n/a*	**0.0169±0.0125**	0.0064±0.0069	0.0110±0.0135	0.0044±0.0064	**0.0125±0.0105**
Roa12	0.0089±0.2271	**−**0.0349±0.4704	**−**0.2255±0.2783	0.0235±0.3098	**−**0.0712±0.2242	**−**0.3492±0.3668
Roa12*Dens12	*n/a*	**−**0.2070±0.4483	0.1763±0.2523	**−**0.4128±0.5183	0.0641±0.1103	**0.4890±0.3816**
Roa34	0.0267±0.0332	**0.0818±0.0628**	0.0255±0.0356	**−**0.0127±0.0484	**−**0.0054±0.0354	**0.1249±0.0342**
Roa34*Dens34	*n/a*	**0.0160±0.0151**	0.0018±0.0109	**0.0203±0.0131**	**0.0179±0.0071**	**0.0072±0.0063**
Dvar12	**0.0914±0.0517**	**−**0.0670±0.0761	**−0.0732±0.0324**	**−**0.0409±0.0450	0.0346±0.0439	**−**0.0016±0.0593
Dvar34	**−**0.0777±0.0815	**−0.1696±0.1107**	**−0.1636±0.0478**	**−0.0925±0.0642**	**−0.1284±0.0664**	**−**0.0600±0.0922

* Confidence intervals can be obtained by adding and subtracting the ±95% CI value to its associated β value.

Informative variables were identified with the 95% CI (i.e. not overlapping zero) when available (if not, noted as ‘*n/a*’) and are identified in bold letters.

**Table 5:** Coefficient estimates (ß) and 95% confidence intervals (95% CI) of the independent variables of the most parsimonious models explaining caribou movements for 49 females in Saguenay – Lac-Saint-Jean (Québec, Canada) between 2004 and 2010 at night.

Variable	Night					
	Spring	Calving	Summer	Rut	Early winter	Late winter
	ß±95% CI*	ß±95% CI*	ß±95% CI*	ß±95% CI*	ß±95% CI*	ß±95% CI*
ElevVar	**−0.0102±0.0033**	**−0.0086±0.0052**	**−0.0041±0.0023**	**−0.0055±0.0029**	**−0.0050±0.0019**	**−0.0097±0.0029**
ElevMoy	0.0039±0.0059	0.0039±0.0090	**0.0095±0.0041**	**0.0060±0.0052**	0.0032±0.0034	**0.0056±0.0051**
Cut05	1.2378±1.5403	*n/a*	0.6482±0.7445	0.9528±0.9577	1.2933±1.3812	**2.6660±1.6883**
Cut05^2^	**−**1.2213±1.4777	*n/a*	**−**0.2734±0.7139	**−**0.6522±0.9238	**−1.6411±1.4286**	**−2.8620±1.7355**
Cut620	**−**0.1977±0.7111	*n/a*	0.0425±0.7690	**−0.7297±0.7053**	**−**0.0571±0.3763	**−0.6579±0.6106**
Cut620^2^	0.3239±0.6602	*n/a*	**0.7780±0.7063**	**1.0794±0.6454**	**−**0.3348±0.3698	0.3919±0.5894
Regen	**−**1.2164±1.2973	*n/a*	**−1.3725±0.7122**	**−0.9673±0.9149**	**−0.6440±0.6337**	**−**0.0650±0.9651
Regen^2^	1.2743±1.3013	*n/a*	**1.3992±0.7031**	0.9266±0.9348	0.4053±0.6393	**−**0.3721±0.9928
Cross_05_	0.0022±0.0748	**−**0.0524±0.1268	**−**0.0379±0.0662	**−**0.0439±0.0772	**−**0.0432±0.0904	**−**0.0350±0.0639
Cross_05_ *Dens_05_	*n/a*	**0.0215±0.0171**	**0.0131±0.0105**	0.0056±0.0122	**−**0.0015±0.0144	**0.0231±0.0143**
Cross_620_	**−**0.0414±0.0426	0.0065±0.0629	**−**0.0489±0.0745	0.0490±0.0514	0.0225±0.0274	0.0009±0.0367
Cross_620_ *Dens_620_	*n/a*	**0.0104±0.0103**	**−**0.0041±0.0107	**−**0.0009±0.0079	**−**0.0010±0.0027	**0.0061±0.0043**
Cross_RGN_	**−0.1524±0.1031**	**−0.2606±0.1889**	**−0.0803±0.0738**	**−0.1040±0.0967**	**−0.1960±0.0745**	**−0.3086±0.1235**
Cross_RGN_ *Dens_RGN_	*n/a*	0.0231±0.0284	0.0075±0.0109	**0.0196±0.0160**	**0.0157±0.0067**	0.0123±0.0168
Roa12	**−**0.1137±0.5139	*n/a*	*n/a*	**−**0.2058±0.5734	**−**0.0141±0.2459	**−1.0641±0.6100**
Roa12*Dens12	*n/a*	*n/a*	*n/a*	0.2284±0.4220	**−**0.1039±0.1593	0.3172±0.3305
Roa34	**0.0981±0.0549**	*n/a*	*n/a*	**−0.0720±0.0717**	**−**0.0364±0.0434	**0.1152±0.0472**
Roa34*Dens34	*n/a*	*n/a*	*n/a*	**0.0138±0.0134**	**0.0220±0.0078**	0.0082±0.0095
Dvar12	0.0813±0.0989	*n/a*	*n/a*	0.0583±0.0780	**0.0900±0.0499**	**0.1064±0.0809**
Dvar34	**−0.2057±0.1557**	*n/a*	*n/a*	**−0.1775±0.1060**	**−0.1501±0.0681**	**−0.1219±0.1164**

* Confidence intervals can be obtained by adding and subtracting the ±95% CI value to its associated β value.

Informative variables were identified with the 95% CI (i.e. not overlapping zero) when available (if not, noted as ‘*n/a*’) and are identified in bold letters.

Females generally avoided crossing major roads, except during the rut at night. Furthermore, individuals were likely to move toward major roads only during the winter periods and spring at night and dusk/dawn. Conversely, caribou preferentially crossed minor roads for all periods except for the rut and early winter, while individuals nonetheless tended to move away from minor roads throughout all periods.

### Impacts of Landscape Context on Step Selection

**Figure 2:**
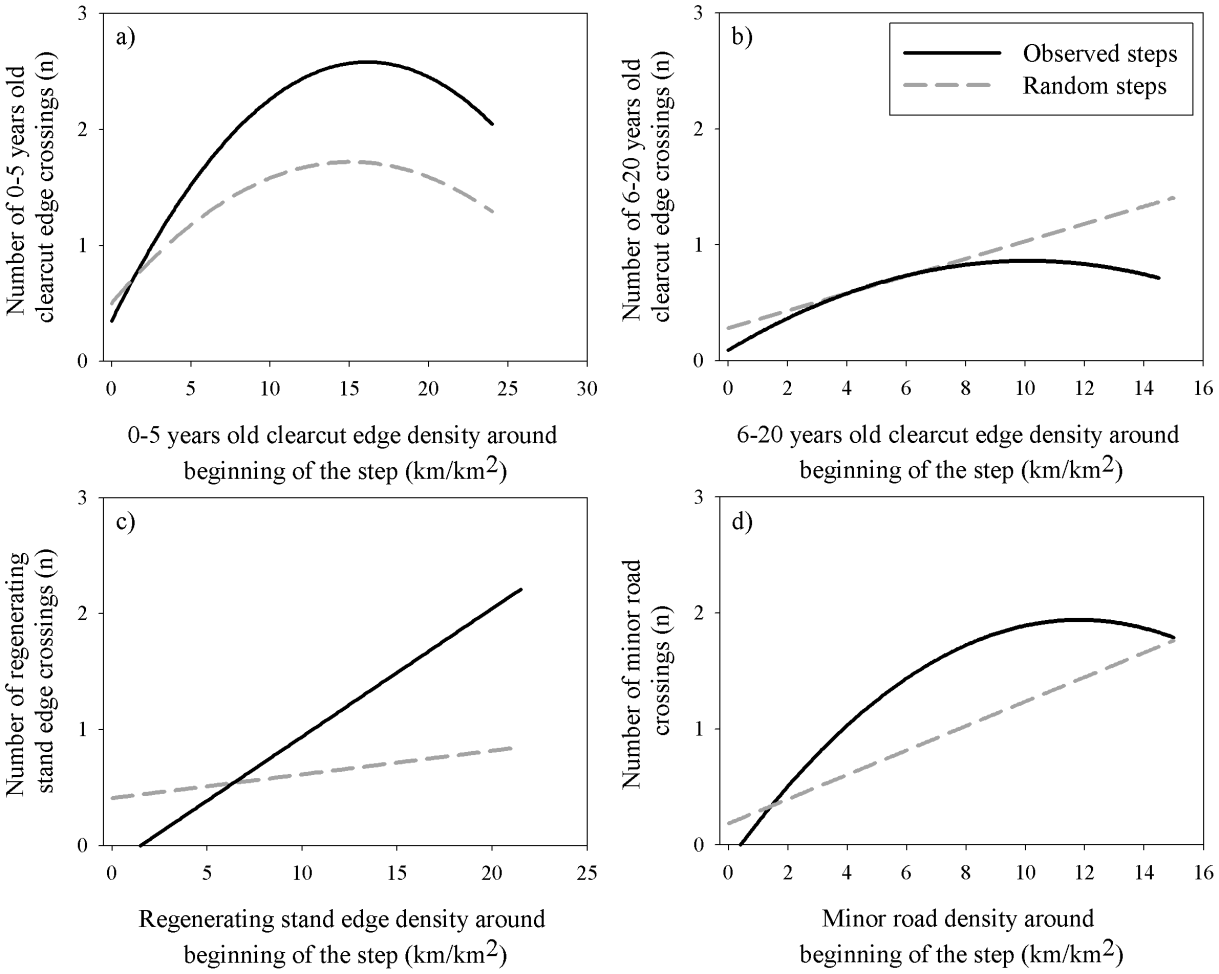
Number of crossing events. a) 0–5 years old clearcut edge crossings during calving at night, b) 6–20 years old clearcut edge crossings during summer at dusk/dawn, c) regenerating stand edge crossings during calving at dusk/dawn and d) minor road crossings during early winter at dusk/dawn as a function of their respective edge density around the beginning of the step. The figures were obtained by fitting a curve on the mean number of crossings per steps for individual caribou within intervals of 0.5 km/km^2^ ranging from 0 to the maximum observed density values, compared to the random steps used in the SSF. We chose four representative examples of typical significant interactions obtained through our analysis (see Tables 3–4–5).

The local context in which females moved influenced their decision to cross clearcut edges and roads for most of the periods considered. Females typically avoided crossing clearcut edges and roads when these features were found at low densities, yet subsequently increased their crossing rates over what would be randomly expected as densities around the beginning of the step increased (Fig. 2a–c–d). In certain instances, however, females rather elected to avoid crossing clearcut edges and roads regardless of the density in which they were located (Fig. 2b). Context was almost always important for major and minor roads, while it seemed to be important mostly during spring, calving and the winter periods for clearcut edges (Tables 3–5).

## Discussion

Caribou are thought to orient their habitat use hierarchically to minimize the impacts of the most limiting factors (e.g. predation) at coarse spatiotemporal scales in order to attend to biological requirements at finer scales [4]. Certain populations nevertheless inhabit heavily altered habitats where the representation of habitats associated with greater uncertainty is such that individuals are also confronted with limiting factors at finer scales [30]. Under this scenario, we investigated the impacts of disturbances on the movements of woodland caribou in highly managed landscapes. We found that although disturbances were essentially avoided, females nonetheless regularly moved through or in close proximity to roads and clearcuts, modifying their behaviour when doing so, confirming yet refocusing hypotheses A and B. Our results also further show that individuals modulate their response to disturbances on a daily and annual basis, especially in periods of significance for caribou ecology, supporting hypothesis C. The increased use of disturbed habitats noted during certain period was however unanticipated and expanded the scope of hypothesis C. We believe that these results could reflect an ability to adapt behavioural decisions temporally to compromise between risk avoidance and forage requirements to optimize their respective efficiency [6], [11], [13], [38]–[39].

### Daily and Annual Response to Disturbances

Female caribou did not respond consistently to the different types of clearcuts. Females were gradually less likely to be found within clearcuts as stands aged, suggesting a temporal evolution of the likelihood of female visiting clearcuts. Moose – and incidentally wolf – densities increase only once young cutovers are regenerating [50]–[51]. Risks of encounter with predators may thus be lower in younger clearcuts [26], increasing the likelihood of caribou using those younger, regenerating habitats [36], [46]. Such habitats are nonetheless associated with increased human [12], [16] and predator activity [26], [28]–[29], which may explain that females preferred using young clearcuts only in combination with locally available alternative habitats [14]. Furthermore, as regeneration will inevitably replace young disturbances, any benefits would be temporary until those young habitats are colonized by predators and their alternative preys.

Females also modified their response to disturbances annually. Avoidance of disturbances seemed to be predominant during periods of greater vulnerability for calves (i.e. calving) and adults (i.e. early and late winter). While calves are very susceptible to opportunistic black bear predation the first weeks following birth [29], [52], adults seem to be more vulnerable to gray wolf predation during the winter as their diet becomes primarily ungulate-based [53]. Specifically, wolves were found to seek caribou habitat during dusk/dawn and at night [54]. Clearcut edges and roads also impacted caribou in periods of greater vulnerability. Edges are known to alter species interactions [55] and may be particularly significant for caribou as edges are used by moose [56] and wolves [57]–[58]. This increase in interactions in the boundary between natural and disturbed habitats could thus explain the distinct avoidance of regenerating stand edges, as observed in other ungulate species [12]. Likewise, roads and edges can be used by alternative prey species, predators and humans, increasing predation risk or disturbance stimuli for caribou [59]. Wolves in particular increase their use of roads during winter, especially at dusk/dawn and at night [54], and encounter rates between caribou and wolves have been found to increase during that period [59]. We believe that as these linear features are associated with increased mortality risk, an increase in their density could then be expected to have important impacts on the survival of female and calves and ultimately lead to population level consequences.

Conversely, individual caribou increased their use of open habitats mainly during the spring, summer and rut periods, along with an increase in the use of 0–5 year-old clearcuts during late winter. This could potentially be explained by forage requirements superseding risk avoidance during those periods. Foraging opportunities become scarcer as winter progresses [3], [60], followed by periods of low body condition in spring and summer that are particularly significant for parturient females [35]. Individuals must therefore adapt their foraging activities during those critical periods and the abundance in shrub cover found in clearcuts in our system has been previously discussed as providing complementary alternative forage for caribou in winter [30]. The green plants available in clearcuts during the snow-free periods could also be used similarly [6], [61]. Contrary to our expectations, females tended to move towards major roads at night through the winter periods. This response may however be expected from individuals accessing open habitats in our study area as they are close to roads, a pattern also observed for reindeer in Sweden relative to trails [43].

Interestingly, female caribou predominantly avoided disturbances during the day. As discussed, although disturbances may offer advantages to caribou during certain annual periods, they are nonetheless associated with increased presence of both alternative prey [25] and predators [26], especially during the day. On the other hand, crepuscular and nocturnal activities of females were not as heavily affected by disturbances, with individuals increasing their use of 6–20 year-old during the summer and the rut. These daily variations in response to disturbances may then reflect a decrease in habitat uncertainty. Such a daily pattern of habitat use is also supported by comparable time-dependant habitat use demonstrated for other ungulate species [6], [12], [38]–[39], [62].

### Influence of Local Context on Movements

The landscape context was found to impact caribou movements, especially during periods of greater vulnerability. Females preferentially increasing or decreasing their crossing rates suggest a vigilance-relocation response potentially related to the risk associated with local disturbance levels, a behavioural adjustment previously noted in elk [12]. As individuals typically avoid crossing clearcut edges and roads, a local increase in the presence of such features can be expected to impose greater alertness on individuals [9] and influence habitat selection patterns [11], [13]–[14]. Increased crossing rates may thus reflect relocation movements in an effort to access more secure areas [11], increasing the likelihood of edge and road crossings [10], [12]. Conversely, the decrease could reflect a state of heightened vigilance compelling individuals to remain within risky habitats for a longer period of time [9]. While increased use of edges and roads could lead to greater predation risk [58]–[59], females that are decreasing their crossing rates may become trapped in sub-optimal habitats and disrupt crucial biological activities [1]. Such a response could have dire consequences for individual survival [63] and seems analogous to responses exhibited at coarser scales, with individuals decreasing space use as disturbance levels increase over certain thresholds [31]. Alongside further local increases in disturbances, females trapped in sub-optimal habitats could ultimately be forced to spend more time foraging and less time assessing risk as they become energetically depleted [9].

## Conclusions

We showed that woodland caribou modify their fine-scale movements temporally in response to disturbances, potentially balancing daily and annual forage requirements with risk avoidance. We also highlighted the importance of considering daily periods when studying behaviour, a common pitfall in habitat selection studies [e.g. 4,7,14,28,30]. The failure to consider daily patterns of habitat use may obscure behaviours like diurnal avoidance and nocturnal use through data aggregation, and potentially fail to detect relevant ecological processes. Additionally, we found that individuals modified their movements when locally confronted with higher disturbance levels, a novel demonstration of synergy between hierarchical spatial scales when characterizing habitat selection and space use patterns. Combined, these two findings seem to indicate that increased disturbance levels in the boreal forest may compel caribou to respond to limiting factors at gradually finer scales and potentially trap them in suboptimal habitats. We know that black bear predation on calves can be particularly problematic in areas of intensive forest management [52]. Additionally, current management practices may increase local caribou densities [64] and co-occurrence probabilities with wolves during the winter period [28], alongside a potential adaptation of wolves to hunt caribou during those periods [54]. It thus seems that predation risk and anthropogenic disturbances act synergistically and impact individual vulnerability, ultimately affecting populations through decreases in reproductive output and survival [1]. Our study contributes to improve our understanding of the effects of landscape heterogeneity on animal movement by covering a large array of habitat disturbances that could have significant impacts on demography [34]. Indeed, proportions of clearcuts within our study area (Portneuf: 41%; Piraube: 15%) fall within range of established levels known to impose detrimental physiological stress (>36%; [15]) and decreased recruitment rates (>35%; [20]), so we could expect further increases in disturbance levels to jeopardize long-term caribou persistence for future generations.
